# Glutathione Status and the Renal Elimination of Inorganic Mercury in the *Mrp2^−/−^* Mouse

**DOI:** 10.1371/journal.pone.0073559

**Published:** 2013-09-05

**Authors:** Christy C. Bridges, Lucy Joshee, Jeroen J. M. W. van den Heuvel, Frans G. M. Russel, Rudolfs K. Zalups

**Affiliations:** 1 Mercer University School of Medicine, Division of Basic Medical Sciences, Macon, Georgia, United States of America; 2 Department of Pharmacology and Toxicology, Radboud University Nijmegen Medical Centre, Nijmegen Centre for Molecular Life Sciences, Nijmegen, The Netherlands; University of California, Merced, United States of America

## Abstract

Multidrug resistance-associated proteins (MRP) 2 and 4 are localized in proximal tubular epithelial cells and participate in the renal elimination of xenobiotics. MRP2 has also been implicated in the renal and hepatic elimination of mercury. The current study tested the hypothesis that MRP2 and MRP4 are involved in renal and hepatic handling of inorganic mercury (Hg^2+^). We examined the disposition of Hg^2+^ in *Mrp2^−/−^* mice and assessed the transport of mercuric conjugates in inside-out membrane vesicles containing human MRP4. Since MRP2 has been shown to utilize glutathione (GSH) for transport of select substrates, we examined renal concentrations of GSH and cysteine and the expression of glutamate cysteine ligase (GCL) in *Mrp2^−/−^* and FVB mice. The effect of Hg^2+^ exposure on renal GSH levels was also assessed in these mice. Our data suggest that MRP2, but not MRP4, is involved in proximal tubular export of Hg^2+^. In addition, GSH levels are greater in *Mrp2^−/−^* mice and exposure to Hg^2+^ reduced renal levels of GSH. Expression of GCL was also altered in *Mrp2^−/−^* mice under normal conditions and following exposure to HgCl_2_. This study provides important novel data regarding the transport of Hg^2+^ and the effect of Hg^2+^ exposure on GSH levels.

## Introduction

The multidrug resistance-associated protein 2 (Mrp2) has been implicated in the cellular export of various endobiotics and xenobiotics, including chemotherapeutic agents and heavy metals such as arsenic [1], [2], platinum [2], [3], cadmium [4], [5], and mercury [2], [6]–[8]. In our previously published studies, we utilized TR^−^ rats to implicate Mrp2 in the proximal tubular elimination of inorganic mercury (Hg^2+^). TR^−^ rats are spontaneous mutants that lack functional Mrp2 and since other proteins have been shown to be altered significantly in these rats, we have chosen, in the current study, to assess the role of Mrp2 in the corporal disposition and handling of Hg^2+^ in a targeted Mrp2 knockout model, i.e., the *Mrp2^−/−^* mouse. Therefore, the purpose of the current study was to: 1) test the hypothesis that Mrp2 is involved in the transport of mercuric species; 2) test the hypothesis that GSH status and biosynthesis is altered by exposure to mercury. Although these studies will be carried out in a manner similar to that used in our previous studies, the current studies using *Mrp2^−/−^* mice are novel and offer several advantages over the use of TR^−^ rats. First, the use of a genetically engineered knockout mouse reduces the possibility that the expression of other genes will be affected as a consequence of the genetic modification. Secondly, the toxicology and toxicokinetics of Hg^2+^ varies considerably between mice and rats (Bridges, unpublished data) and the current studies will provide important novel information regarding the handling of inorganic mercury (Hg^2+^) in mice. Thirdly, the mouse is one of the most popular research models used currently and thus, it is important to fully characterize the handling of Hg^2+^ by these animals. The data obtained from the current study may be utilized for comparison to data obtained from future studies utilizing mouse models. Although a portion of data obtained from the current study tends to confirm our previous findings from TR^−^ rats, the use of *Mrp2^−/−^* mice to study the disposition and handling of Hg^2+^ is novel and significant.

The results of our previous *in vitro* studies led us to suggest that another transport mechanism, in addition to Mrp2, was involved in the proximal tubular elimination of mercuric ions, particularly when Hg^2+^ was conjugated to 2,3-bis(sulfanyl)propane-1-sulfonic acid (formerly known as 2,3-dimercaptopropane-1-sulfonic acid; DMPS). One possible candidate for this transport is Mrp4, which is localized in the apical plasma membrane of proximal tubular cells [9]. Owing to its localization and its ability to transport a broad range of substrates, Mrp4 also appears to play an important role in the renal elimination of endobiotics and xenobiotics. Prior to the present study, no data have been published regarding the ability of Mrp4 to transport Hg^2+^. In the current study, we utilized inside-out membrane vesicles containing human MRP4 in order to assess the ability of this carrier to mediate the transport of DMPS-*S*-conjugates of Hg^2+^.

It is well-established that Hg^2+^ is a powerful oxidant that accumulates rapidly in proximal tubular cells [10], [11] and it has been shown than exposure of rats to organic forms of mercury (e.g., methylmercury) can induce the expression of glutamate cysteine ligase (GCL), the rate-limiting enzyme in the synthesis of glutathione (GSH). This induction leads to an increase in the synthesis of glutathione (GSH) [12]–[15]. In contrast, little is known about the effects of inorganic mercury on the individual steps in the biosynthetic pathway of GSH. Published studies indicate that GSH levels increase following exposure to inorganic mercury [16], but the mechanism behind this increase has yet to be characterized. In addition, tissue levels of GSH and the biosynthesis of GSH has not been characterized in *Mrp2^−/−^* mice. Therefore, in the current study, we assessed aspects of renal GSH biosynthesis and the effects of mercury on this metabolic pathway in these mice. To our knowledge, the present study not only represents the first report of corporal disposition of Hg^2+^ in *Mrp2^−/−^* mice, but also it represents the first assessment of GSH metabolism and the effects of Hg^2+^ on GSH synthesis in the kidneys of these mice.

## Methods

### Ethics Statement

All experiments utilizing animals were approved by the Mercer University Institutional Animal Care and Use Committee (IACUC, Permit A1108009). Animals were handled in accordance with the NIH Guide for the Care and Use of Laboratory Animals.

### Animals

Breeder pairs of *Mrp2^−/−^* mice [17] were obtained from Taconic (Germantown, NY) and were mated in our animal care facility. Friend Virus B (FVB) mice, which were used as control mice, were obtained from Charles River Laboratories (Wilmington, MA) and mated in our facility. All animals were provided a commercial laboratory diet (Tekland 6% rodent diet, Harlan Laboratories) and water *ad libitum* throughout all aspects of experimentation.

### Manufacture of [^203^Hg]

The protocol for manufacturing radioactive mercury ([^203^Hg]) has been described previously [18], [19]. Briefly, three milligrams of mercuric oxide were sealed in quartz tubing and were irradiated by neutron activation for 4 weeks at the Missouri University Research Reactor (MURR) facility. After irradiation, the mercuric oxide was dissolved in 1 N HCl. The radioactivity of the solution was determined using an Ion Chamber survey meter (Fluke Biomedical, Everett, WA). The specific activities of the [^203^Hg] ranged from 6–12 mCi/mg.

### Injection of Mice with [^203^Hg]

FVB and *Mrp2^−/−^* mice, weighing 25–30 g, were injected intraperitoneally (i.p.) with a non-toxic dose (0.5 µmol ⋅ kg*^−^* ^1^ ⋅ 8 mL*^−^* ^1^) of HgCl_2_ (designed to deliver 1 µCi [^203^Hg] per mouse). This dose is equivalent to 135.75 µg ⋅ kg*^−^* ^1^ HgCl_2_. Mice were dosed according to weight with a volume of 8 mL ⋅ kg*^−^* ^1^. Each mouse strain was divided randomly into two groups of four mice each, following which mice were placed individually in metabolic cages. Twenty-four and twenty-eight hours after injection with HgCl_2_, one group of *Mrp^−/−^* mice and one group of FVB mice were injected i.p. with 100 mg ⋅ kg*^−^* ^1^ ⋅ 8 mL*^−^* ^1^ DMPS. At the same time, the remaining *Mrp2^−/−^* and FVB mice were injected i.p. with 8 mL ⋅ kg*^−^* ^1^ normal saline. Forty-eight hours after injection with HgCl_2_, mice were injected with a mixture of ketamine (70 mg ⋅ kg*^−^* ^1^) and xylazine (30 mg ⋅ kg*^−^* ^1^) and were sacrificed via exsanguination 48 hours. Organs were harvested for determination of mercury content.

### Collection of Tissues, Organs, Urine and Feces

At the time of sacrifice, mice were anesthetized with an intraperitoneal overdose of ketamine (70 mg ⋅ kg*^−^* ^1^) and xylazine (30 mg ⋅ kg*^−^* ^1^). A 0.5-mL sample of blood was obtained from the inferior vena cava. Approximately 0.1 mL was placed in a polystyrene tube for estimation of [^203^Hg] content. The remaining 0.4 mL was placed in a blood separation tube in order to separate plasma from the cellular components of blood. Total blood volume was estimated as 6% of total body weight [20].

Each kidney was removed, weighed and cut in half along a transverse plane. A 3-mm transverse slice of the left kidney was utilized for separation of cortex (S1 and S2 proximal tubular segments) and outer stripe of outer medulla (S3 proximal tubular segments). Each zone of the kidney was weighed and placed in a separate polystyrene tube for estimation of [^203^Hg] content. In addition, a small slice of left kidney was frozen in liquid nitrogen for future RNA isolation. One-half of the right kidney was frozen in liquid nitrogen for future HPLC analyses. The liver was then excised, weighed, and a 1-g section of liver was removed for determination of [^203^Hg] content.

Urine and feces were collected for 24 h and 48 h after injection with HgCl_2_. There were no significant differences in urine volume or fecal mass among animals. Urine from each animal was mixed and a 0.1-mL sample was weighed and placed in a polystyrene tube for estimation of [^203^Hg] content. All of the feces excreted by each animal during each 24-h period were counted to determine the total fecal content of [^203^Hg] excreted by each animal. The content of [^203^Hg] in each sample was determined using standard isotopic methods and by counting in a Wallac Wizard 3 automatic gamma counter (Perkin Elmer, Boston, MA).

### High Performance Liquid Chromatography (HPLC)

Concentrations of GSH and Cys in renal tissues from *Mrp2^−/−^* and FVB mice injected intraperitoneally with 0.5 µmol ⋅ kg*^−^* ^1^ ⋅ 8 mL*^−^* ^1^ or 10 µmol ⋅ kg*^−^* ^1^ ⋅ 8 mL*^−^* ^1^ HgCl_2_ were measured using HPLC. Tissues were frozen in liquid nitrogen, pulverized and then homogenized in 10% (v/v) perchloric acid. Samples were centrifuged for 10 min at 12,000×g and the acid-soluble fraction was utilized for analyses. An aliquot of the acid-soluble fraction was combined with bathophenanthroline disulfonic acid (BPDS) and γ-glutamyl glutamate in 70% perchloric acid. [21] From this mixture, a 0.5-mL aliquot was removed and derivatized using iodoacetate and 1-fluoro-2,4-dinitrobenzene. Separation of derivatives was achieved with a Shimadzu SCL-10A solvent delivery system fitted with a µBondapak amine-equivalent 10 µm column (9.6 mm x 10 cm; ES Industries, West Berlin, NJ) using a methanol-acetate mobile phase with gradient elution. Derivatives were detected at 365 nm using a Shimadzu SPD-10A detector. Quantification was based on known standards of GSH or Cys. To control for inter-sample variation, peak areas were normalized to the internal standard (γ-glutamyl glutamate).

### Vesicular Transport Assays

Inside-out membrane vesicles were prepared from human embryonic kidney (HEK293) cells expressing human MRP2 or MRP4 in the Bac-to-Bac vector as described previously [22]. Control vesicles were prepared from HEK293 cells mock-transduced with the same vector, containing the enhanced yellow fluorescent protein (EYFP) gene. MRP2 and MRP4 transport activity was validated by measuring the uptake of [^3^-H]-methotrexate as described previously [22], [23]. Vesicular transport assays were carried out using a rapid filtration method according to a published protocol [22], [23]. Briefly, DMPS-*S*-conjugates of Hg^2+^ were formed by mixing 5 µM [^203^Hg] with 12.5 µM DMPS in incubation buffer (250 mM sucrose, 10 mM Tris/HCl, pH 7.4) supplemented with 10 mM MgCl_2_, 10 mM creatine phosphate and 100 µg/ml creatine phosphokinase in the presence of 4 mM ATP or AMP). Incubation buffer containing DMPS-*S*-conjugates of Hg^2+^ was added to vesicle mixture (7.5 µg protein) and incubated for 30 seconds at 37°C. Following incubation, ice-cold buffer containing 1 mM DMPS (to remove bound Hg) was added and each sample was filtered through a Multiscreen plate (0.45 µm; Millipore, Billarica, MA). Filters were removed and radioactivity contained on filter was determined using liquid scintillation spectroscopy.

In order to assess ATP-dependent transport, the amount of [^203^Hg] associated with vesicles in the presence of AMP was subtracted from that in the presence of ATP.

### RNA Isolation and Real-time PCR Analyses

Kidneys from *Mrp2−/−* and FVB mice injected intraperitoneally with 0.5 µmol ⋅ kg*^−^* ^1^ ⋅ 8 mL*^−^* ^1^ or 10 µmol ⋅ kg*^−^* ^1^ ⋅ 8 mL*^−^* ^1^ HgCl_2_ were isolated at the time of animal sacrifice. A 3-mm transverse slice was obtained from the central region of the kidney and was frozen immediately in liquid nitrogen. At the time of RNA isolation, frozen kidney slices were pulverized with a mortar and pestle. TRIzol Reagent (Life Technologies, Grand Island, NY) was added to the ground kidney and RNA was extracted according to the manufacturer’s protocol.

Reverse transcription of 1 µg of RNA was carried out using reverse transcriptase and random hexamers (Life Technologies). Samples were subjected to the following conditions: 10 min at 25°C; 60 min at 42°C; 4 min at 95°C. For real-time PCR analyses, 2 µl of the reverse transcriptase reaction were utilized and samples were subjected to the following conditions: 2 min at 50°C; 10 min at 95°C; 1 min at 60°C for 40 cycles. Analysis of glutamate cysteine ligase (GCL, formerly known as γ-glutamyl cysteine synthetase) was performed with an ABI Prism 7000 detection system using a Gene Expression Assay (Mm0000514996_m1, Life Technologies) designed using a published GCL sequence (NM_008129.3). Glyceraldehyde 3-phosphate dehydrogenase (Gapdh) was used as a reference gene. The Gene Expression Assay for Gapdh (Mm99999915_g1) was designed using a published sequence (NM_008084.2).

### Data Analysis

Data from animal experiments are presented as percent of administered dose of mercury or percent of administered dose of mercury per gram of tissue (in order to account for potential differences in body mass between animals). Data were then analyzed with the Kolmogorov-Smirnov test for normality and Levene’s test for homogeneity of variances. Following these tests, data were analyzed using a one-way analysis of variance (ANOVA), a 2-way ANOVA, or a 2×4 ANOVA to assess differences among the means. When statistically significant *F*-values were obtained with ANOVA, the data were analyzed using Tukey’s *post hoc* multiple comparison test. A *p*-value of <0.05 was considered statistically significant. Each group of animals contained three or four mice. All data are expressed as mean ± standard error.

## Results

### Disposition of Mercuric Ions in Mouse Tissues

**Figure 1:**
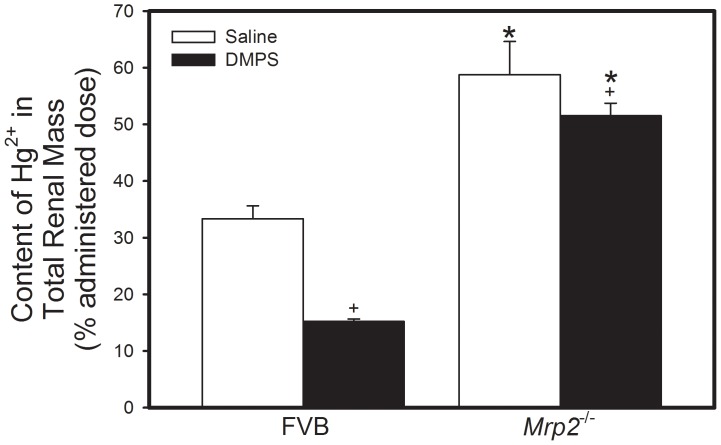
Content of Mercury in the Total Renal Mass of *Mrp2^−/−^* and FVB Mice. *Mrp2^−/−^* and FVB mice were injected intraperitoneally with a dose of 0.5 µmol ⋅ kg*^−^* ^1^ ⋅ 8 mL*^−^* ^1^ HgCl_2_. Kidneys were harvested 48 h after injection with HgCl_2_. Data represent mean ± SE of kidneys from four mice. *, significantly different from mean of corresponding FVB mice (p<0.05). +, significantly different from mean of mice from same strain treated with saline (p<0.05).

Approximately 30% of a 0.5 µmol ⋅ kg*^−^* ^1^ dose of Hg^2+^ was detected in the total renal mass of FVB mice 48 h after exposure to HgCl_2_ and treated subsequently with saline (Figure 1). In contrast, nearly 60% of the administered dose of Hg^2+^ was detected in the total renal mass of corresponding *Mrp2^−/−^* mice. Treatment of FVB mice with DMPS (100 mg ⋅ kg*^−^* ^1^ ⋅ 8 mL*^−^* ^1^) reduced the renal burden of Hg^2+^ by over 50% (Figure 1). When *Mrp2^−/−^* mice were treated with the same dose of DMPS, the renal burden of Hg^2+^ was found to be significantly lower than that in *Mrp2^−/−^* mice treated with saline.

**Figure 2:**
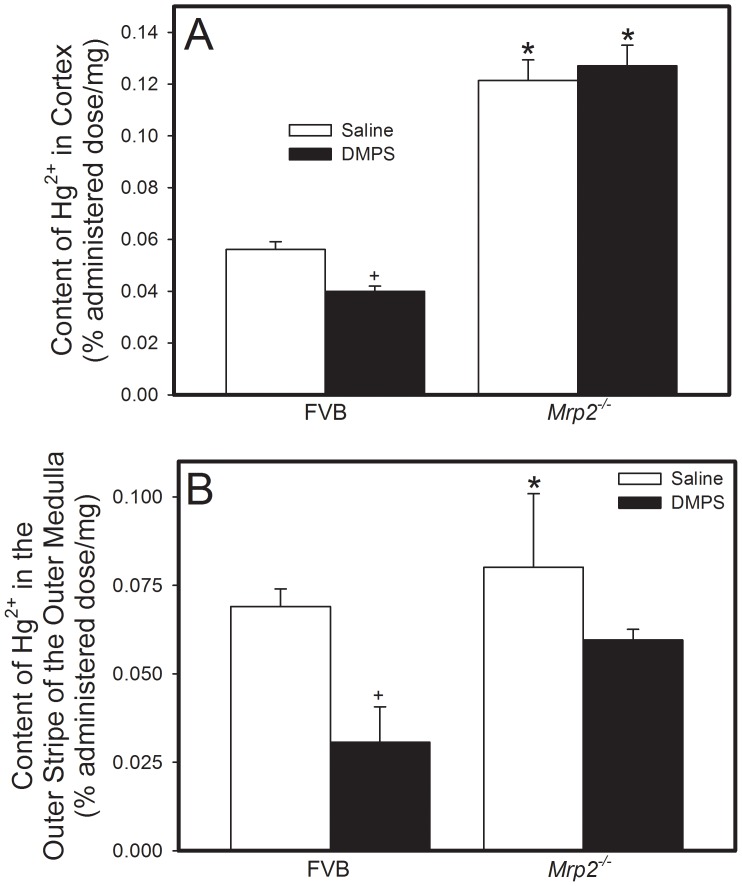
Content of Mercury in the Renal Zones of *Mrp2^−/−^* and FVB Mice. *Mrp2^−/−^* and FVB mice were injected intraperitoneally with a dose of 0.5 µmol ⋅ kg*^−^* ^1^ ⋅ 8 mL*^−^* ^1^ HgCl_2_. Samples from cortex (A) or outer stripe of outer medulla (B) were harvested 48 h after injection with HgCl_2_. Data are presented as percent administered dose per milligram of tissue in order to account for differences in sample weight. Data represent mean ± SE of four mice. *, significantly different from mean of corresponding FVB mice (p<0.05). +, significantly different from mean of mice from same strain treated with saline (p<0.05).

The content of mercuric ions in the renal cortex of *Mrp2^−/−^* mice exposed to HgCl_2_ and treated subsequently with saline was approximately twofold greater than that of corresponding FVB mice (Figure 2A). Treatment with DMPS significantly reduced the cortical content of Hg^2+^ in FVB but not in *Mrp2^−/−^* mice. Similarly, the content of Hg^2+^ in the outer stripe of the outer medulla of kidneys from *Mrp2^−/−^* mice was significantly greater than that from corresponding FVB mice (Figure 2B). Treatment with DMPS significantly reduced the content of Hg^2+^ in the outer stripe of the outer medulla in both strains of mice.

**Figure 3:**
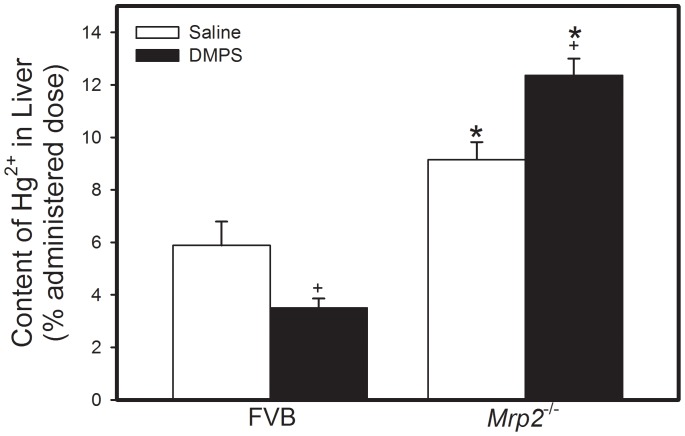
Content of Mercury in Liver of *Mrp2^−/−^* and FVB Mice. *Mrp2^−/−^* and FVB mice were injected intraperitoneally with a dose of 0.5 µmol ⋅ kg*^−^* ^1^ ⋅ 8 mL*^−^* ^1^ HgCl_2_. Samples of liver were harvested 48 h after injection with HgCl_2_. Data represent mean ± SE of liver samples from four mice. *, significantly different from mean of corresponding FVB mice (p<0.05). +, significantly different from mean of mice from same strain treated with saline (p<0.05).

The hepatic burden of Hg^2+^ in *Mrp2^−/−^* mice exposed to HgCl_2_ and treated subsequently with saline was significantly greater than that in corresponding FVB mice (Figure 3). Treatment with DMPS significantly reduced the hepatic burden of Hg^2+^ in FVB mice. In contrast, when *Mrp2^−/−^* mice were treated with DMPS, the amount of Hg^2+^ detected in liver increased significantly (Figure 3).

Approximately 1.3 percent of the administered dose of Hg^2+^ was detected in blood of both groups of *Mrp2−/−* mice (saline and DMPS). The amount of Hg^2+^ detected in blood of FVB mice treated with saline was also approximately 1.3 percent of the administered dose. In contrast, the hematologic burden of Hg^2+^ in FVB mice exposed to HgCl_2_ and treated subsequently with DMPS was approximately 0.7 percent of the administered dose, which was significantly different from that of all remaining groups of mice. Approximately 75% of Hg^2+^ was associated with the cellular components of blood while the remaining 25% was detected in plasma. This pattern of distribution was similar in all groups of mice.

**Figure 4:**
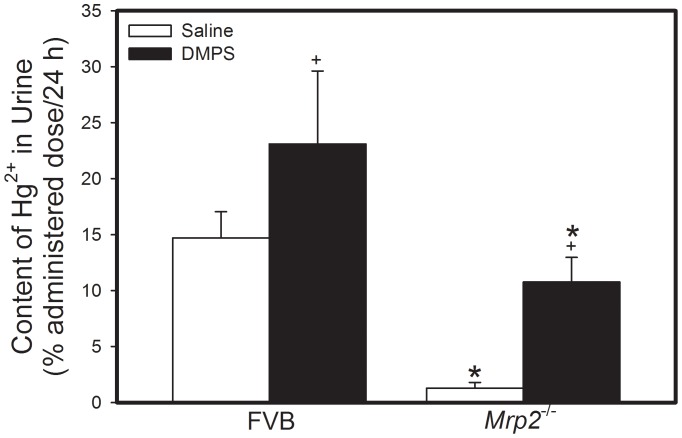
Content of Mercury in Urine of *Mrp2^−/−^* and FVB Mice. *Mrp2^−/−^* and FVB mice were injected intraperitoneally with a dose of 0.5 µmol ⋅ kg*^−^* ^1^ ⋅ 8 mL*^−^* ^1^ HgCl_2_. Urine was collected 24 h and 48 h after injection with HgCl_2_. Data represent mean ± SE of urine collected from four mice. *, significantly different from mean of corresponding FVB mice (p<0.05). +, significantly different from mean of mice from same strain treated with saline (p<0.05).

**Figure 5:**
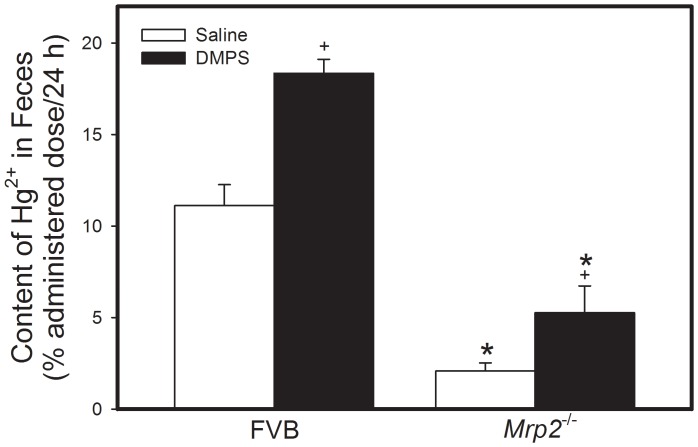
Content of Mercury in Feces of *Mrp2^−/−^* and FVB Mice. *Mrp2^−/−^* and FVB mice were injected intraperitoneally with a dose of 0.5 µmol ⋅ kg*^−^* ^1^ ⋅ 8 mL*^−^* ^1^. Feces were collected 24 h and 48 h after injection with HgCl_2_. Data represent mean ± SE of feces collected from four mice. *, significantly different from mean of corresponding FVB mice (p<0.05). +, significantly different from mean of mice from same strain treated with saline (p<0.05).

The amount of Hg^2+^ in urine collected over a 48 h-period from FVB mice exposed to HgCl_2_ followed by treatment with saline was significantly greater than that from corresponding *Mrp2^−/−^* mice (Figure 4). Treatment with DMPS significantly increased the urinary excretion of Hg^2+^ in both, FVB and *Mrp2^−/−^* mice. Similarly, fecal excretion of Hg^2+^ from FVB mice exposed to HgCl_2_ and treated with saline was significantly greater than that by corresponding *Mrp2^−/−^* mice (Figure 5). Treatment with DMPS significantly increased fecal excretion of Hg^2+^ by both strains of mice.

### Transport of DMPS-S-conjugates of Hg^2+^ in Membrane Vesicles

**Figure 6:**
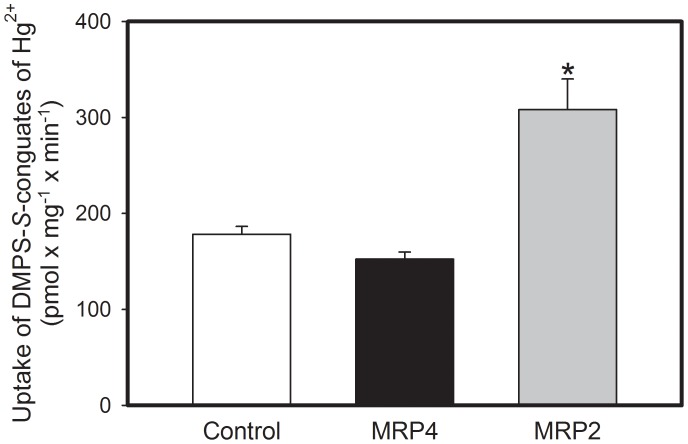
Transport of DMPS-*S*-conjugates of Hg^2+^ into Inside-out Membrane Vesicles. Inside-out membrane vesicles expressing human MRP2, MRP4, or EYFP (control) were exposed to DMPS-*S*-conjugates of HgCl_2_ for 30 s at 37°C. Data represent mean ± SE of four replicates. +, significantly different from mean of control vesicles (p<0.05).

DMPS-S-conjugates of Hg^2+^ were formed by incubation of 5 µM HgCl_2_ with 12.5 µM DMPS at room temperature for five minutes. Transport of DMPS-*S*-conjugates of Hg^2+^ was measured in control vesicles and in vesicles containing MRP2 or MRP4 (Figure 6). ATP-dependent uptake of Hg^2+^ was significantly greater in MRP2-containing vesicles than in control vesicles. In contrast, the amount of Hg^2+^ associated with MRP4-containing vesicles was not significantly different from that of control vesicles.

### HPLC Analyses of GSH and Cysteine in Kidneys from FVB and Mrp2^−/−^ Mice

**Figure 7:**
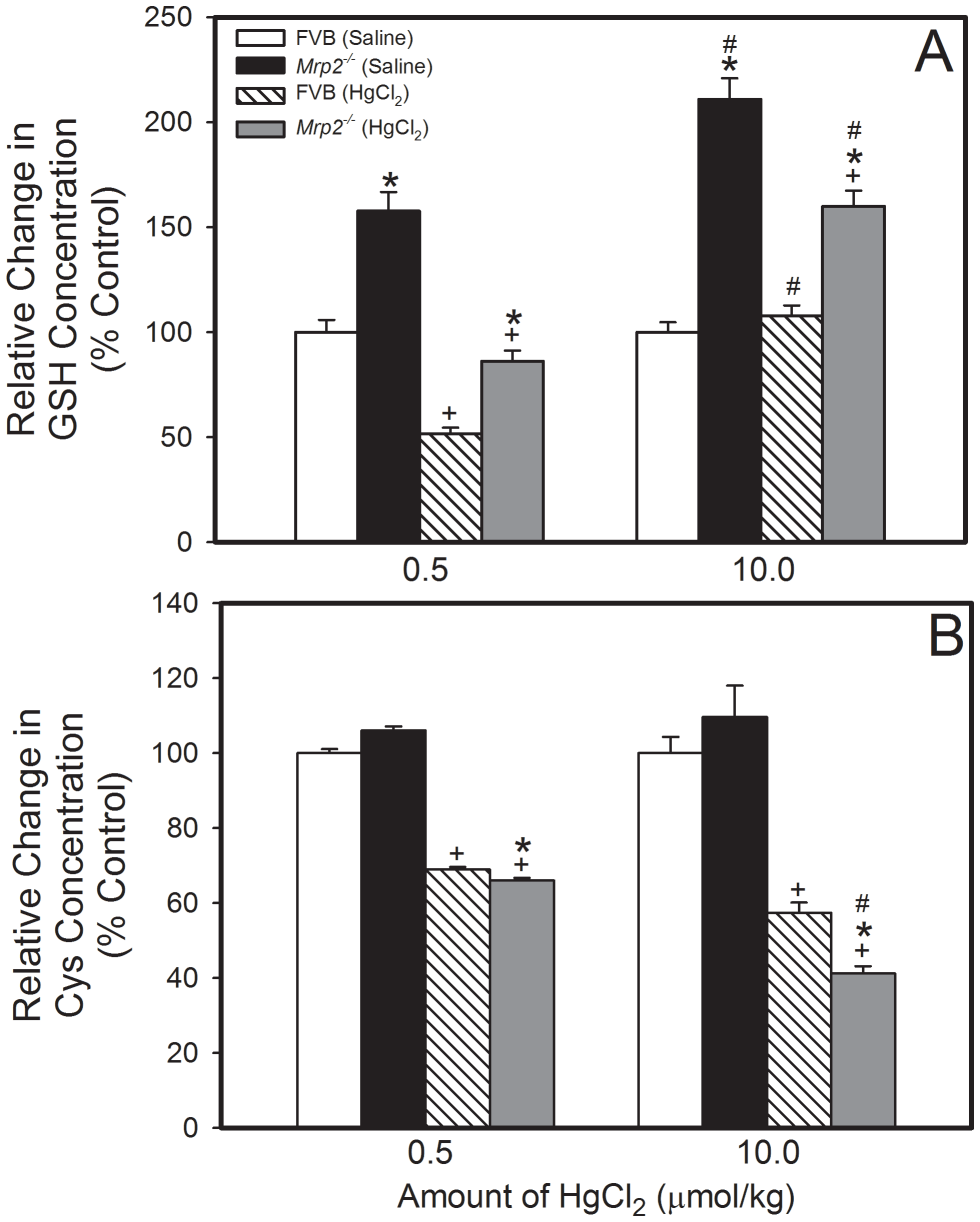
HPLC Analysis of Renal Glutathione and Cysteine Concentration in *Mrp2^−/−^* and FVB Mice. HPLC analyses of renal GSH (A) or cysteine (Cys) (B) were carried out on 3-mm slices of kidneys from *Mrp2^−/−^* and FVB mice exposed to saline, 0.5 µmol ⋅ kg*^−^* ^1^ ⋅ 8 mL*^−^* ^1^, or 10 µmol ⋅ kg*^−^* ^1^ ⋅ 8 mL*^−^* ^1^ HgCl_2_. Renal levels of GSH or Cys were normalized to known GSH or Cys standards, respectively. Data represent mean ± SE of nine samples from three different animals. *, significantly different from the mean of corresponding FVB mice (p<0.05). +, significantly different from the mean of the same strain of mouse treated with saline (p<0.05). #, significantly different from the mean of the corresponding group of mice treated with 0.5 µmol kg*^−^* ^1^ HgCl_2_.

HPLC analyses of GSH in kidneys from FVB and *Mrp2^−/−^* mice (not exposed to HgCl_2_) indicate that the concentration of GSH was significantly greater in kidneys from *Mrp2^−/−^* mice than that in kidneys from corresponding FVB mice (Figure 7A). Interestingly, exposure of mice to 0.5 µmol ⋅ kg*^−^* ^1^ HgCl_2_ reduced the concentration of GSH in both strains of mice by approximately 50%. The renal concentration of GSH remained greater in *Mrp2^−/−^* mice than in FVB mice. Interestingly, treatment of FVB mice with 10 µmol ⋅ kg*^−^* ^1^ HgCl_2_ did not significantly alter the renal concentration of GSH. In contrast, when *Mrp2^−/−^* mice were exposed to 10 µmol ⋅ kg*^−^* ^1^⋅ 8 mL*^−^* ^1^ HgCl_2_, the renal concentration of GSH was reduced by only 25% (Figure 7A).

The renal concentration of Cys in unexposed *Mrp2^−/−^* mice was not significantly different from that in corresponding FVB mice (Figure 7B). When mice were treated with 0.5 µmol ⋅ kg*^−^* ^1^ HgCl_2_, the renal concentration of Cys, in each strain, was reduced by approximately 35% (Figure 7B). Interestingly, after treatment with 10 µmol ⋅ kg*^−^* ^1^ HgCl_2_, the renal concentration of Cys was reduced by approximately 60% in *Mrp2^−/−^* mice and by approximately 40% in FVB mice (Figure 7B).

### Real-time PCR Analyses of Glutamate Cysteine Ligase (GCL)

**Figure 8:**
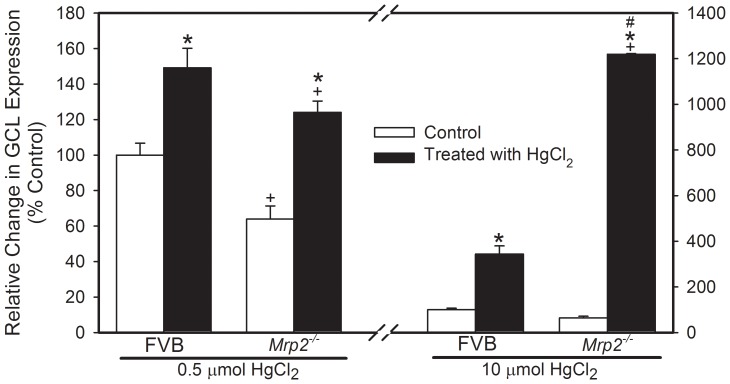
RT-PCR Analysis of Glutamate Cysteine Ligase (GCL) Expression in *Mrp2^−/−^* and FVB Mice. Real-time PCR analyses were carried out on 3-mm slices of kidneys from *Mrp2^−/−^* and FVB mice treated with saline, 0.5 µmol ⋅ kg*^−^* ^1^ ⋅ 8 mL*^−^* ^1^, or 10 µmol kg*^−^* ^1^ 8 mL*^−^* ^1^ HgCl_2_. Samples were normalized with glyceraldehyde-3-phosphate dehydrogenase (Gapdh). Data represent mean ± SE of nine samples from three different animals. *, significantly different from mean of corresponding FVB mice (p<0.05). +, significantly different from the mean of the same strain of mice treated with saline (p<0.05). #, significantly different from the mean of the corresponding group of mice treated with 0.5 µmol ⋅ kg*^−^* ^1^ HgCl_2_.

The renal expression of GCL (formally γ-glutamylcysteine synthetase) was significantly lower in *Mrp2^−/−^* mice not exposed to HgCl_2_ than in corresponding FVB mice (Figure 8). Following exposure of FVB mice to 0.5 µmol ⋅ kg*^−^* ^1^ HgCl_2_, the expression of GCL increased by approximately 50%. In *Mrp2^−/−^* mice, exposure to 0.5 µmol ⋅ kg*^−^* ^1^ HgCl_2_ resulted in a two-fold increase in the expression of GCL. When FVB mice were exposed with 10 µmol ⋅ kg*^−^* ^1^ HgCl_2_, the expression of GCL increased nearly 3.5-fold. Treatment of *Mrp2^−/−^* mice with 10 µmol ⋅ kg*^−^* ^1^ HgCl_2_, increased the expression of GCL by approximately 19-fold.

## Discussion

The current dispositional studies in *Mrp2^−/−^* mice demonstrate that within the kidneys, the content of Hg^2+^ in the renal cortex of *Mrp2^−/−^* mice was twofold greater than that in corresponding FVB mice. In the outer stripe of the outer medulla, the content of Hg^2+^ was about 25% greater in *Mrp2^−/−^* mice than in FVB mice. Interestingly, in kidneys of *Mrp2^−/−^* mice, the amount of Hg^2+^ in the renal cortex was twofold greater than that in the outer stripe of the outer medulla. This finding is likely due to preferential accumulation of mercuric ions in S1 and S2 proximal tubular segments [11], which are the primary sites of Mrp2 expression and localization [24]. In addition, urinary excretion of mercuric ions was lower in *Mrp2^−/−^* mice than in control mice, which supports the idea that, in the absence of Mrp2, mercuric ions are retained within proximal tubular epithelial cells and consequently, are not excreted in urine. The present findings, combined with previous data from TR^−^ rats [6], [7], [25], provide substantial, cross-species evidence indicating that Mrp2 plays a significant role in the export of mercuric ions from proximal tubular cells. In addition, these data represent the first characterization of mercury handling in Mrp2−/− mice and indicate that *these* mice are a viable model for studies on the role of Mrp2 in the handling and disposition of Hg^2+^.

A role for Mrp2 in the hepatobiliary excretion of mercuric ions was initially proposed by Sugawara and colleagues [8]. They showed that mercuric ions were retained in the liver of Mrp2-deficient Eisai hyperbilirubinemic (EHB) rats. Similarly, our data from TR^−^ rats also suggest that Mrp2 is involved in the hepatobiliary export of mercuric ions [6], [7], [25]. In the current study, the hepatic burden of mercury was significantly greater in *Mrp2^−/−^* mice than in FVB mice. In addition, fecal excretion of mercuric ions was lower in *Mrp2^−/−^* mice than in FVB mice. Together, these data provide significant support for the hypothesis that Mrp2 plays an important role in the elimination of mercuric ions from hepatocytes.

When *Mrp2^−/−^* mice were treated with the chelator/complexing agent, DMPS, subsequent to exposure to Hg^2+^ the renal burden of Hg^2+^ in both, control and *Mrp2^−/−^* mice, was reduced significantly. We have hypothesized previously that DMPS forms complexes with mercuric ions intracellularly and that these complexes are transported out of cells via Mrp2. Because treatment of *Mrp2^-/−^* mice with DMPS was able to reduce the renal burden of mercury to some extent, we conclude that another transport mechanism, in addition to Mrp2, is involved in the DMPS-mediated export of mercuric ions from proximal tubules. A putative candidate for this process is Mrp4. Owing to similarities in substrate specificity of Mrp2 and Mrp4 [26] and the fact that Mrp4 is localized in the apical membrane of proximal tubular epithelial cells [9], we postulated that Mrp4 may also play a role in the proximal tubular export of mercuric ions. Surprisingly, the results of our membrane vesicle experiments suggest that DMPS-*S*-conjugates of Hg^2+^ are not substrates of MRP4. In contrast, DMPS-*S*-conjugates of Hg^2+^ were found to be transported readily in inside-out membrane vesicles containing MRP2. Although whole-animal dispositional findings from our laboratory indicate that an additional transporter, other than MRP2, plays a role in the export of mercuric ions from proximal tubular cells, the current *in vitro* findings suggest that this carrier is likely not MRP4. Other possible candidates that may participate in the export of DMPS-*S*-conjugates of Hg^2+^ include the organic anion transporter 5 (OAT5; *SLC22A10*) and/or the multidrug resistance protein 1 (MDR1/P-glycoprotein; *ABCB1*), both of which are localized in the luminal membrane of proximal tubular cells [27]–[29].

Interestingly, when *Mrp2^−/−^* mice were treated with DMPS, the hepatic burden of Hg increased significantly. We believe that this increase may be due to binding of DMPS to mercuric ions in other tissues and subsequent mobilization of the mercuric ions. Given the role of the liver in drug processing and other metabolic processes, it is reasonable to suggest that mobilized DMPS-Hg complexes are delivered to the liver in an attempt to process and eliminate this toxicant.

MRP2 has been shown to transport GSH and GSH-*S*-conjguates, as well as utilize it as a co-factor for the transport of certain other substrates. In addition, GSH is involved in numerous cellular processes including drug metabolism, maintenance of cellular redox status, and cellular stress responses, such as that which occurs following exposure to Hg^2+^ [16], [30]. Because GSH plays an important role in cellular responses following exposure to Hg^2+^, we assessed the renal levels of GSH in *Mrp2^−/−^* and FVB mice treated with saline or HgCl_2_ in order to 1) determine if differences in GSH synthesis exist between these two strains of mice (that can be attributed to the absence of Mrp2) and 2) to examine the effects of HgCl_2_ exposure on GSH status. It is logical to postulate that in the absence of Mrp2, substrates of this transporter, including GSH, may accumulate within cells. Therefore, it was not surprising that renal levels of GSH were greater in *Mrp2^−/−^* mice than in FVB mice. In order to determine if the increased levels of GSH were due to increased synthesis or the lack of GSH export, we examined mRNA levels of glutamate cysteine ligase (GCL), which is the rate-limiting enzyme required for the synthesis of GSH. Interestingly, renal expression of GCL was significantly lower in *Mrp2^−/−^* mice than in FVB mice, suggesting that the increased levels of GSH in *Mrp2^−/−^* mice may be due to the lack of GSH export rather than an increase in GSH synthesis. The reduced expression of GCL in *Mrp2^−/−^* mice is most likely the result of GSH-mediated feedback inhibition wherein increased levels of GSH inhibit the expression of GCL [31].

Given the oxidative nature of Hg^2+^ we chose to examine the effects of exposure to this metal on renal GSH levels and biosynthesis in *Mrp2^−/−^* and FVB mice To our knowledge, this study represents the first examination of the effects of Hg^2+^ exposure on renal GSH levels and biosynthesis in these mouse models. Exposure of animals to a non-toxic dose (0.5 µmol ⋅ kg*^−^* ^1^) of HgCl_2_ led to a reduction of renal GSH levels, possibly due to Hg^2+^-induced depletion of intracellular GSH stores or inhibition of GSH biosynthesis. Under these conditions, the renal concentration of Cys, which is the rate-limiting substrate of GSH synthesis, was also reduced, perhaps due to its utilization for GSH biosynthesis. Indeed, renal expression of GCL was increased twofold in both, FVB and *Mrp2^−/−^* mice following exposure to HgCl_2,_ suggesting an attempt to increase the synthesis of GSH in proximal tubular cells.

Previous studies in rats have shown that increasing in the dose of Hg^2+^ leads to enhanced renal cellular concentrations of GSH [16]. Therefore, in the current study, FVB and *Mrp2^−/−^* mice were also exposed to a greater, albeit non-toxic dose of HgCl_2_ (10 µmol ⋅ kg*^−^* ^1^). In these mice, the overall renal levels of GSH were greater than those in mice exposed to a non-toxic (0.5 µmol ⋅ kg*^−^* ^1^) dose of HgCl_2_. When we compared the renal levels of GSH in FVB and *Mrp2^−/−^* mice exposed to saline with those in mice exposed to 10 µmol ⋅ kg*^−^* ^1^ HgCl_2_, we found that exposure to a larger dose of HgCl_2_ did not alter the overall renal levels of GSH in FVB mice, but did reduce the renal concentration of GSH in *Mrp2^−/−^* mice. This finding may be related to the observation that the baseline expression of GCL was lower in *Mrp2^−/−^* mice than in FVB mice (due to feedback inhibition). Therefore, FVB mice may be able to synthesize GSH at a faster rate than *Mrp2^−/−^* mice. Also, following exposure to 10 µmol ⋅ kg*^−^* ^1^ HgCl_2_, renal levels of Cys were reduced significantly in both strains of mice, possibly due to enhanced utilization for GSH synthesis.

The expression of GCL in each strain of mouse corresponded with measured levels of GSH and Cys. As expected, expression of GCL increased significantly in both strains of mice after exposure to a 10 µmol ⋅ kg*^−^* ^1^ dose of HgCl_2_, suggesting that synthesis of GSH was initiated. The expression of GCL was significantly greater in mice exposed to a dose of 10 µmol ⋅ kg*^−^* ^1^ HgCl_2_ than that observed following exposure to 0.5 µmol ⋅ kg*^−^* ^1^ HgCl_2_, suggesting that the need to synthesize GSH was greater in animals treated with a higher dose of HgCl_2_.

In summary, the present data from a targeted knockout mouse model (e.g., *Mrp2^−/−^*) provide substantive novel evidence supporting a role of Mrp2 in the renal and hepatic elimination of mercuric ions. In addition, we provide new data from inside-out membrane vesicles containing human MRP2 or MRP4 suggesting that MRP2, but not MRP4, plays a significant role in DMPS-mediated elimination of mercuric ions from proximal tubules. The present study is also the first to show differences in renal GSH biosynthesis and GCL expression between *Mrp2^−/−^* and FVB mice and the ability of HgCl_2_ to alter these parameters. Overall, this study presents novel findings that provide valuable information regarding the transport of mercuric species and the effects of these mercuric species on select cellular processes in kidneys.
